# In *Arabidopsis thaliana* Heterosis Level Varies among Individuals in an F_1_ Hybrid Population

**DOI:** 10.3390/plants9040414

**Published:** 2020-03-27

**Authors:** Hasan Mehraj, Takahiro Kawanabe, Motoki Shimizu, Naomi Miyaji, Ayasha Akter, Elizabeth S. Dennis, Ryo Fujimoto

**Affiliations:** 1 Graduate School of Agricultural Science, Kobe University, Rokkodai, Nada-ku, Kobe 657-8501, Japan; hmehraj34@stu.kobe-u.ac.jp (H.M.); 162a318a@stu.kobe-u.ac.jp (N.M.); 154a371a@stu.kobe-u.ac.jp (A.A.); 2 School of Agriculture, Tokai University, Toroku, Higashi-ku, Kumamoto 862-8652, Japan; 3 Iwate Biotechnology Research Center, Narita, Kitakami, Iwate 024-0003, Japan; m-shimizu@ibrc.or.jp; 4 Department of Horticulture, Bangladesh Agricultural University, Mymensingh 2202, Bangladesh; 5 CSIRO Agriculture and Food, Canberra, ACT 2601, Australia; Liz.Dennis@csiro.au; 6 University of Technology, Sydney, PO Box 123, Broadway, NSW 2007, Australia

**Keywords:** heterosis, hybrid vigour, transcriptome

## Abstract

Heterosis or hybrid vigour is a phenomenon in which hybrid progeny exhibit superior yield and biomass to parental lines and has been used to breed F_1_ hybrid cultivars in many crops. A similar level of heterosis in all F_1_ individuals is expected as they are genetically identical. However, we found variation in rosette size in individual F_1_ plants from a cross between C24 and Columbia-0 accessions of *Arabidopsis thaliana*. Big-sized F_1_ plants had 26.1% larger leaf area in the first and second leaves than medium-sized F_1_ plants at 14 days after sowing in spite of the identical genetic background. We identified differentially expressed genes between big- and medium-sized F_1_ plants by microarray; genes involved in the category of stress response were overrepresented. We made transgenic plants overexpressing 21 genes, which were differentially expressed between the two size classes, and some lines had increased plant size at 14 or 21 days after sowing but not at all time points during development. Change of expression levels in stress-responsive genes among individual F_1_ plants could generate the variation in plant size of individual F_1_ plants in *A. thaliana*.

## 1. Introduction

Heterosis or hybrid vigour is the superior performance of F_1_ (heterozygous) plants relative to their inbred (homozygous) parental lines. In the process of plant breeding, the phenomenon of heterosis has been exploited in various crops and vegetables because of its effect on yield or stress tolerance [1]. Hybrid breeding has been remarkably successful starting with maize [2,3], but the molecular mechanism of heterosis remains unknown. Several genetic models have been hypothesized for the explanation of heterosis [1,4,5,6]. The dominance model explains that heterosis is due to the complementation of deleterious recessive alleles by favourable dominant alleles at multiple loci. The overdominance model argues that the heterozygous state leads to superior performance of hybrids to either homozygous condition. The epistasis model is that interaction of favourable alleles at different loci results in heterosis. Epigenetic modifications are considered to also contribute to heterosis; interactions between parental epigenetic states in the two sets of chromosomes in hybrids play a role in heterosis [5,7,8]. Mutations in the chromatin remodeler *Decreased in DNA methylation 1* (*DDM1*) lead to lower levels of heterosis, supporting a role for epigenetic contributions to heterosis [8].

The concept that the superior performance of hybrid is caused by establishment of more favourable gene expression levels relative to the parental lines has been considered [9]. The transcriptome profile has been compared between hybrids and parental lines in a number of heterotic hybrids of maize, rice, and *Arabidopsis thaliana* [10,11,12,13,14,15,16,17,18]. Though the majority of genes show an additive gene expression pattern, differentially expressed genes between hybrids and the mid-parent value (MPV), termed non-additively expressed genes, are detected [1]. In some studies, non-additively expressed genes involved in specific functional categories have been suggested to play a role in the heterosis phenotype, while there are reports showing that the majority of non-additively expressed genes are not associated with any specific categories [10,11,12,13,14,15,16,17,18,19]. 

In addition to crops and vegetables, *A. thaliana* also shows substantial heterosis in vegetative biomass in particular parental combinations [20,21,22,23,24,25], and several approaches such as quantitative trait locus (QTL) analysis, transcriptome, metabolome, small RNAome, and epigenome analysis have been used to identify genes and mechanisms that may be important for heterosis [1,8]. In the hybrid between C24 and Columbia-0 (Col) accessions of *A. thaliana*, heterosis is obvious at early developmental stages in increased cotyledon area at a few days after sowing. Larger cotyledon size generates an increase in photosynthetic capacity, suggesting that this increased photosynthetic capacity in hybrids may cause the maintenance and/or magnification of heterosis at later developmental stages [14]. A similar phenotype at early developmental stages has been observed in other parental combinations of *A. thaliana* [26]. 

In this study, we found variation in size among individual plants, which were hybrid between the C24 and Col accessions. To identify the genes regulating the altered plant size in individuals with the same genetic background, we compared the transcriptome profile between big- and medium-sized F_1_ plants using microarrays. A number of genes showed a higher expression level in the big-sized F_1_ plants than in the medium-sized F_1_ plants; we examined their effect on plant size by overexpression, focusing on genes categorized into ‘transcription factor’. A number of transgenic plants were larger at 14 or 21 days after sowing, suggesting that these genes play a part in the control of plant size.

## 2. Results

### 2.1. Variation of Shoot Size in the F_1_ between C24 and Col

**Figure 1:**
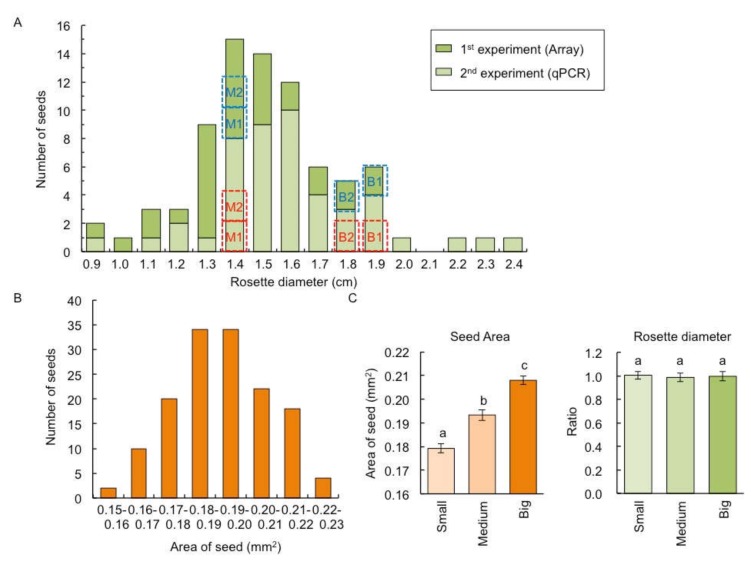
Variation of rosette diameter and seed size among individual F_1_ plants between C24 and Columbia-0 (Col). (**A**) Distribution of rosette diameter among individual F_1_ plants (*n* = 80) at 14 days after sowing (DAS). Dark and light green bars represent plant materials used for microarray analysis and quantitative RT-PCR (qPCR), respectively. Blue and red dotted lines represent the plant size used for microarray and qPCR, respectively. (**B**) Distribution of seed area in F_1_ (*n* = 72). (**C**) The seed area (left panel) and rosette diameter at 14 DAS (right panel) derived from small, medium, and big seed fractions. The ratio of the rosette diameter compared with plants derived from big seed fractions are shown. Data presented are the average and standard error (s.e.) (*n* = 9). Letters above the bars indicate significant differences at *p* < 0.05 (Tukey–Kramer test).

The F_1_ between C24 and Col had a heterosis phenotype in shoots [14,15]. From our previous data [14], we found that the variation of rosette diameter of F_1_ hybrid between C24 and Col was larger than that of parental lines (Figure S1). This study confirmed this phenomenon; among 80 F_1_ plants, shoot size evaluated by rosette diameter at 14 days after sowing (DAS) varied, and the biggest rosette diameter was 2.7 times larger than smallest (Figure 1A). The size of dry seed evaluated by seed area also showed variation within the seventy-two F_1_ seeds, and the seed area of the biggest seed was 1.5 times larger than the smallest (Figure 1B). 

To examine whether the larger shoot size in F_1_ plants is due to larger seed size, we examined the relationship between shoot and seed sizes. There was no difference in rosette diameter at 14 DAS of nine F_1_ plants derived from nine seeds from each of the small, medium, and big seed fractions (Figure 1C), indicating that the difference in seed size is independent of shoot size in the F_1_ population. 

**Figure 2:**
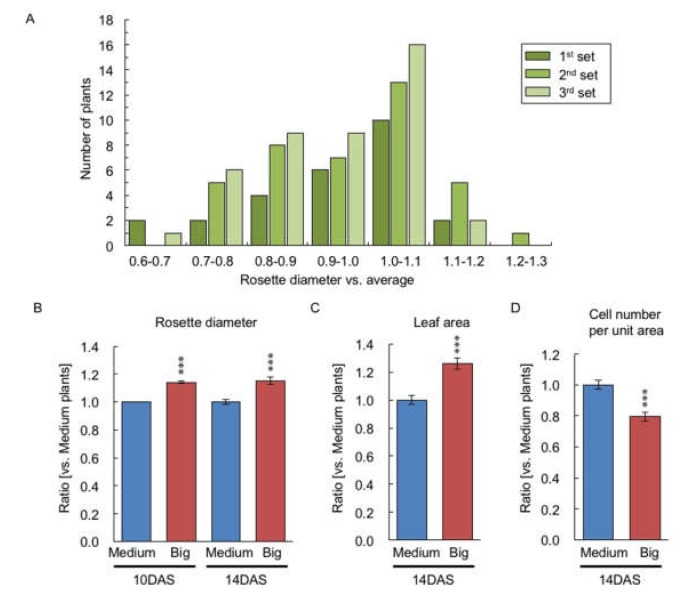
Comparison of the rosette diameter and true leaf area and its cell number per unit area between medium- and big-sized F_1_ plants. (**A**) Distribution of ratio of rosette diameter compared with average of rosette diameter in F_1_ plants (*n* = 103) at 10 DAS. Three replicates were performed represented as first, second, and third sets. (**B**) Ratio of rosette diameter compared with medium-sized F_1_ plants in big-sized F_1_ plants at 10 and 14 DAS. (**C**) Ratio of leaf area of first and second leaves compared with medium-sized F_1_ plants in big-sized F_1_ plants at 14 DAS. (**D**) Ratio of cell number per unit area in the first layer of palisade mesophyll cell compared with medium-sized F_1_ plants in big-sized F_1_ plants at 14 DAS. Data presented are the average and standard error (s.e.) (each, *n* = 22). *** *p* < 0.001 (Student’s *t*-test).

Using twenty-two plants of each of the big-(14.1% larger) and medium-size fractions in rosette diameter at 10 DAS from 103 F_1_ plants (Figure 2A), the rosette diameter, leaf area, and the size of the first layer of palisade mesophyll cell in the first and second leaves were examined at 14 DAS. At 14 DAS, F_1_ plants that were big at 10 DAS retained the larger rosette diameter (15.2%) relative to the medium-sized F_1_ plants (Figure 2B), and the big-sized F_1_ plants had 26.1% larger leaf area in the first and second leaves relative to the medium-sized F_1_ plants (Figure 2C). The big-sized F_1_ plants had a 21.6% reduction in the number of the first layer of palisade mesophyll cells per unit area relative to the medium-sized F_1_ plants at 14 DAS (Figure 2D), indicating that the larger leaf area of the big-sized F_1_ plant is due to the increased cell size.

### 2.2. The Transcriptome Divergence between Big- and Medium-Sized F_1_ Plants

We examined the whole genome transcriptome of big- and medium-sized F_1_ plants at 14 DAS using the Affymetrix, Arabidopsis ATH1 Genome Array using total RNAs from shoots of the biggest (B1, two 1.9 cm rosette diameter plants), the second biggest (B2, two 1.8 cm rosette diameter plants), and two medium-size fractions (M1 and M2, two 1.4 cm rosette diameter plants) of plants (Figure 1A). We did not use small F_1_ plants, as there is a risk that the decrease in plant size is due to disease or failure to thrive. The 441 probe sets showed 1.5-fold difference with 5% false discovery rate (FDR) in expression between B1&B2 and M1&M2 (Table S1). Among differentially expressed genes, 361 (81.9%) probe sets were expressed at a higher level in B1&B2 than in M1&M2 (B1&B2 > M1&M2 expression), and 80 probe-sets showed B1&B2 < M1&M2 expression (Table S1). 

We compared the lists of non-additively expressed genes in aerial tissues between three F_1_ hybrids (C24/Col, C24/Landsberg *erecta* (L*er*), Col/L*er*) and their mid-parent values (MPV) at 15 DAS [17] with our lists of differentially expressed genes (Table 1). About 30% of genes showing B1&B2 > M1&M2 expression overlapped with upregulated genes in C24/Col or C24/L*er* hybrids (Table 1), and these ratios were higher than the expected ratio (chi-square goodness of fit test, *p* < 10^−10^). The 61 genes (16.9%) overlapped with upregulated genes in both C24/Col and C24/L*er* hybrids (Table S2), while 5.8% of genes overlapped with upregulated genes in Col/L*er* hybrids (Table 1). Of genes showing B1&B2 < M1&M2 expression, more genes overlapped with downregulated genes in C24/Col hybrids (31.3%) followed by C24/L*er* (22.5%) and Col/L*er* (13.8%) hybrids (Table 1). The ratios overlapping B1&B2 < M1&M2 expression and downregulated genes in C24/Col and C24/L*er* were also higher than the expected ratio (chi-square goodness of fit test, *p* < 10^−10^).

We also compared our data with lists of differentially expressed genes between C24xCol and *ddm1*C24x*ddm1*Col hybrids or between ColxC24 and *ddm1*Colx*ddm1*C24 hybrids (Table 1), which showed different plant size with the same genetic background [27]. As our material is C24xCol hybrids, we focused on genes differentially expressed in C24xCol and *ddm1*C24x*ddm1*Col hybrids; twenty-five genes showing B1&B2 > M1&M2 expression overlapped with downregulated genes in C24xCol hybrids compared with *ddm1*C24x*ddm1*Col hybrids (Table 1), including *Ethylene responsive element binding factor 5* (*ERF5*), *ERF6*, *ERF105*, *WRKY40*, *DRE BINDING PROTEIN 1B* (*DREB1B*), *Salt-inducible zinc finger 1* (*SZF1*), and *SZF2* (Figure S2). 

### 2.3. Confirmation of Differential Gene Expression by Quantitative RT-PCR

**Figure 3:**
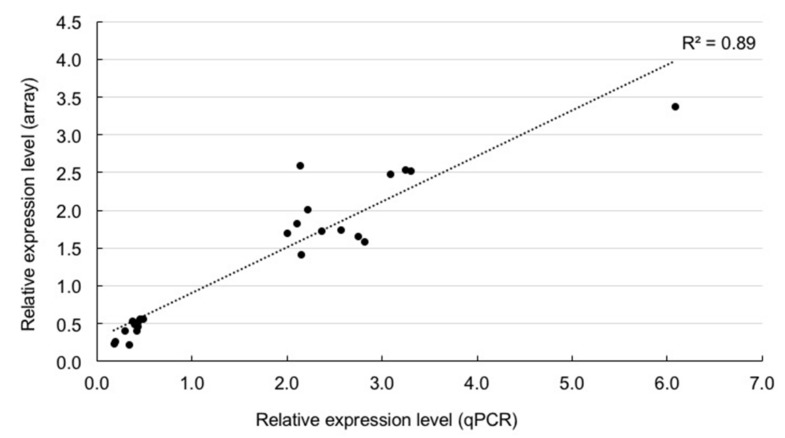
Verification of microarray data by qPCR. Relationship of relative expression levels between qPCR and microarray in 24 differentially expressed genes between big- and medium-sized F_1_ plants.

We confirmed the expression patterns of 24 differentially expressed genes (13 genes, B1&B2 > M1&M2 expression; 11 genes, B1&B2 < M1&M2 expression) using quantitative RT-PCR analysis (qPCR) using another set of big- (B1, two 1.9 cm rosette diameter plants; B2, two 1.8 cm rosette diameter plants) and medium-sized (M1 and M2, two 1.4 cm rosette diameter plants) F_1_ plants (Figure 1A). The relative expression levels between B1&B2 and M1&M2 seen in microarray data were highly correlated with those in qPCR (*r* = 0.94, *p* < 1.00 × 10^-10^) (Figure 3, Table S3). 

### 2.4. Comparison of Gene Expression between Big- and Medium-Sized F_2_ Plants

**Figure 4:**
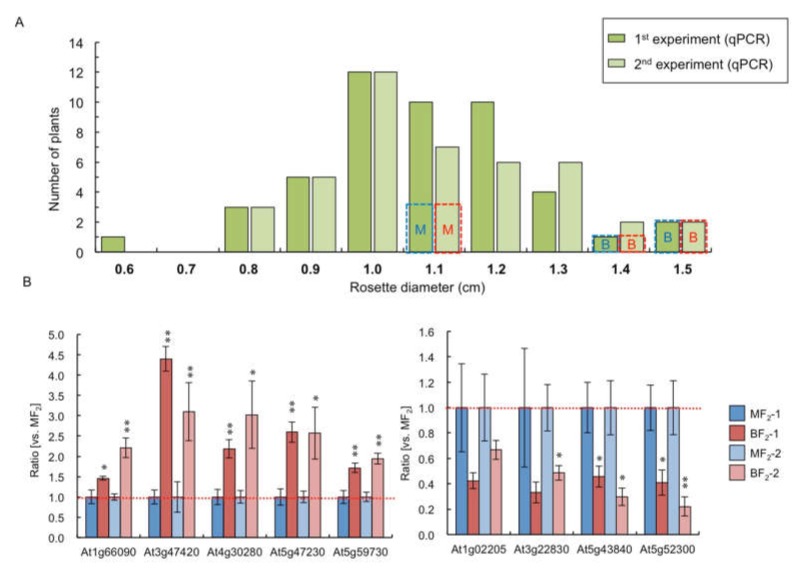
Comparison of the gene expression level between big- and medium-sized F_2_ plants. (**A**) Distribution of rosette diameter at 14 DAS of F_2_ plants. Blue and red dotted lines represent the plant size used for qPCR. (**B**) qPCR using genes that showed B1&B2 > M1&M2 (left panel) and B1&B2 < M1&M2 expression (right panel). The ratio of the expression levels compared with medium-sized F_2_ plants in big-sized F_2_ plants is shown. Data presented are the average and standard error (s.e.) from three biological and experimental replicates. * *p* < 0.05, ** *p* < 0.01 (Student’s *t*-test).

Rosette diameter at 14 DAS varied in F_2_ plants derived from medium- (first) and big- (first) sized F_1_ hybrid between C24 and Col (Figure 4A). Mean size of rosette diameter and distribution of plant size were similar between two F_2_ populations (Figure 4A), suggesting that plant size differences in F_1_ were not inherited in the next generation. We examined whether the same genes showed differential gene expression between big (BF_2_, two 1.5 cm and one 1.4 cm rosette diameter plants) and medium (MF_2_, three 1.1 cm rosette diameter plants) fractions of F_2_ plants as in F_1_ (Figure 4A). The 14 and 11 genes showing larger differences in B1&B2 > M1&M2 and B1&B2 < M1&M2 expression, respectively, were used. In the first F_2_ big fraction, six and four genes showed BF_2_ > MF_2_ and BF_2_ < MF_2_ expression, respectively (Figure 4B, Table S4). In the second F_2_ fraction, nine and four genes showed BF_2_ > MF_2_ and BF_2_ < MF_2_ expression, respectively (Figure 4B, Table S4). Five and two genes showed BF_2_ > MF_2_ and BF_2_ < MF_2_ expression, respectively, in both sets (Figure 4B, Table S4). About 35% (5/14 genes) and 18% (2/11 genes), as in the F_1_, showed a higher or lower expression level in the big-sized F_2_ plants than in the medium-sized F_2_ plants, respectively. 

### 2.5. Classification of the Differentially Expressed Genes between Big- and Medium-Sized F_1_ Plants

**Figure 5:**
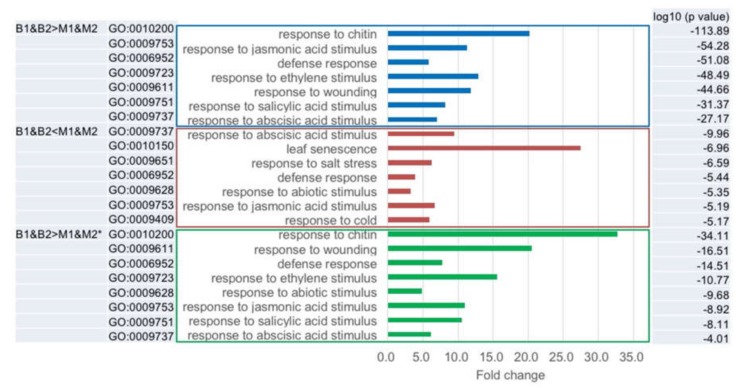
Gene ontology (GO) classification in B1&B2 > M1&M2 and B1&B2 < M1&M2 expressed genes. * represents overrepresented GO categories in B1&B2 > M1&M2 expressed genes and differentially expressed genes in C24/Col and C24/L*er* hybrids compared with MPV.

**Figure 6:**
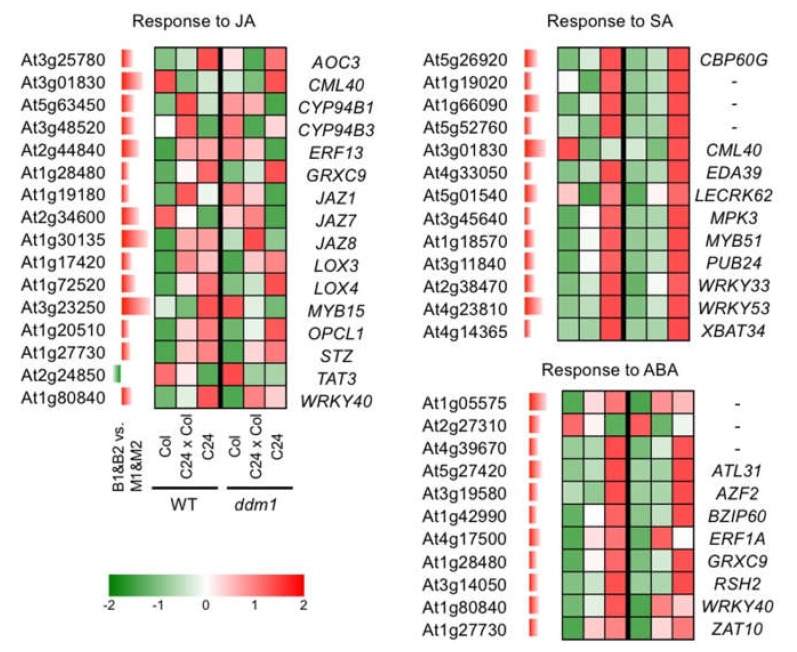
Expression pattern of genes showing B1&B2 > M1&M2 expression. GO terms related to plant hormone response are shown. Expression levels in Col, C24, C24xCol, *ddm1*Col, *ddm1*C24, and *ddm1*C24x*ddm1*Col are derived from [27]. Different red/green colours indicate the fold change (up/down) from the MPV. ABA, abscisic acid; JA, jasmonic acid; SA, salicylic acid.

We categorized the differentially expressed genes between B1&B2 and M1&M2 into gene ontology (GO) cellular component, GO molecular function, and GO biological process (Tables S5 and S6). The categories of ‘Response to abscisic acid stimulus’, ‘Defense response’, and ‘Response to jasmonic acid stimulus’ in GO biological process were overrepresented in genes showing B1&B2 > M1&M2 and B1&B2 < M1&M2 expression (Figure 5 and Figure 6, Tables S5 and S6). In the genes showing B1&B2 > M1&M2 expression, genes categorized into ‘Response to chitin’, ‘Response to ethylene stimulus’, ‘Response to wounding’, and ‘Response to salicylic acid stimulus’ in GO biological process were overrepresented (Figure 5 and Figure 6, Table S5). In the genes showing B1&B2 < M1&M2 expression, genes categorized into ‘Leaf senescence’, ‘Response to salt stress’, ‘Response to abiotic stimulus’, and ‘Response to cold’ in GO biological process were overrepresented (Figure 5 and Figure 6, Table S6). In the 61 genes, which showed B1&B2 > M1&M2 expression and upregulated genes in both C24xCol and C24xL*er* hybrids (Table S2), similar categories to B1&B2 > M1&M2 expressed genes were overrepresented (Figure 5, Table S7). 

### 2.6. Overexpression Resulted in Plant Size Difference

**Figure 7:**
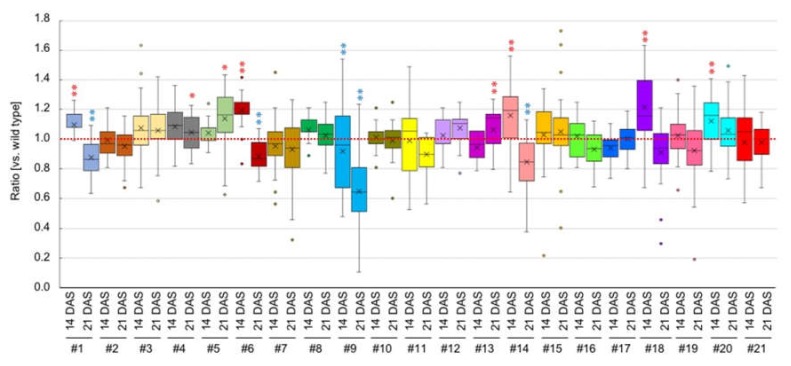
Rosette diameter of overexpressed plants. y-axis shows the ratio of rosette diameter in transgenic plants compared with non-transgenic plants. *p < 0.05, ** *p* < 0.01 (Student’s *t*-test). Blue and red asterisks represent decrease and increase plant size, respectively.

We focused on the B1&B2 > M1&M2 expressed genes, especially transcription factors as they control many biological processes by regulating gene expression. cDNAs from the first methionine to the stop codon of 21 genes were placed under the control of a 35S promoter, and binary vectors were transformed into the wild-type Col accession of *A. thaliana* (Table 2). We obtained more than three independent T_1_ transgenic plants for each gene. Bulked T_2_ seeds were sown on MS medium, and we compared the rosette diameter at 14 and 21 DAS of T_2_ plants with and without transgenes. Twelve of 21 lines showed no difference between transgenic plants and non-transgenic controls at either 14 or 21 DAS (Figure 7, Table 2). In two lines, #18 and #20, the rosette diameter of transgenic plants was larger than that of non-transgenic plants at 14 DAS (Figure 7, Table 2). Three lines, #1, #6, and #14, had larger rosette diameter than non-transgenic plants at 14 DAS, but the rosette diameter was smaller at 21 DAS (Figure 7, Table 2). In three lines, #4, #5, and #13, the rosette diameter in transgenic plants was larger than non-transgenic plants only at 21 DAS (Figure 7, Table 2). A transgenic line, #9, showed a smaller rosette diameter than non-transgenic plants at both 14 and 21 DAS, and plants had pleiotropic seedling size (Figure S3). Although there was no rosette size difference in #19 compared with non-transgenic controls, all plants with transgenes showed narrow and light green leaves (Figure S3). We confirmed the overexpression in some genes compared with control plants (Figure S4).

## 3. Discussion

One of the advantages of F_1_ hybrid cultivars in crops and vegetables is their uniform phenotype, which makes management of cultivation easier [28]. This uniformity of F_1_ hybrid cultivars is considered to be due to the high rate of homozygosity of the parental lines. As *A. thaliana* has a high rate of inbreeding [29], the genetic background in the C24xCol hybrid should be identical in individual plants, and C24xCol hybrids should show a similar level of heterosis in each plant. However, our results showed variation in plant size among individual F_1_ plants; the biggest rosette diameter is more than two times larger than the smallest. This increased leaf area in big-sized F_1_ plants is due to increased cell size. These results suggest the possibility that heterosis level and/or plant size are affected by epigenetic changes because of their identical genetic background. One possibility is that there is epigenetic variation within individual F_1_ plants. Non-additive DNA methylation occurs in the F_1_ by the allelic interaction of different DNA methylation states in parental lines [30,31], and the DNA methylation state might not be uniform in individual F_1_ plants. The epigenetic inbred (epiRIL) lines, which have a difference of DNA methylation level with the same genetic background, show higher divergence of flowering time and plant height compared to wild-type Col [32]. This phenomenon could explain plant size variation in individual F_1_ plants. The other possibility is that epigenetic variation was generated by environmental effects. One candidate is differences of light intensity as in this hybrid, there is increased heterosis under increased light intensity [21]. In our experiments, plants were grown under well-controlled conditions and differences of light intensity between plants were small (150–180 μmol·m^−2^·s^−1^), but this small difference might result in sizes of F_1_ plants varying.

We identified differentially expressed genes between big- and medium-sized F_1_ plants, and approximately 30% of genes showing B1&B2 > M1&M2 expression overlapped with genes upregulated in heterotic F_1_ plants (C24/Col and C24/L*er*) compared with MPV. These genes were categorized into response to wounding, defense response, and response to plant hormones such as ethylene (ET), abscisic acid (ABA), salicylic acid (SA), and jasmonic acid (JA). Transcriptome analysis comparing heterotic F_1_ and MPV suggested several possibilities to explain the heterosis. (1) Decreased expression levels of defence-responsive genes may play an important role in heterosis by reducing energy cost for defence and releasing resource allocation to plant growth [17,24,33]. (2) A reduction in SA concentration with lower expression levels of SA responsive genes is associated with increased biomass [17]. (3) Negative effects of ET on heterosis have been suggested [34]. (4) Delayed senescence could be involved in heterosis at later developmental stages [35]. In our transcriptome data, similar categories were overrepresented in differentially expressed genes between big- and medium-sized F_1_ plants. However, sometimes the direction of change of expression levels does not match between big- vs. medium-sized F_1_ plants and the heterotic F_1_ vs. MPV; i.e., in big-sized F_1_ plants, more defence-responsive genes were upregulated, and higher expression levels in genes involved in response to ET or SA stimulus were found. These results suggest that these genes are involved in determining the plant size, and any further increase in plant size of heterotic F_1_ plants may result from a different expression pattern to non-additive expression.

Loss of DDM1 function showed a decreased heterosis level compared with wild-type F_1_ [27,36]. The 25 genes showing B1&B2 > M1&M2 expression overlapped with downregulated genes in wild-type F_1_ compared with *ddm1* F_1_, i.e., an opposite expression pattern. The relationship between increased/decreased expression level and plant size does not necessarily match in the big-size F_1_ plants and the decreased plant size in *ddm1* F_1_, and changing expression levels of these genes might be involved in the change of plant size.

Heterosis has been suggested to affect ET biosynthesis or signal transduction, and overexpression of the ET biosynthesis gene, *1-aminocyclopropane-1- carboxylate synthase 6* (*ACS6*), eliminated heterosis [34]. In this study, we made transgenic plants overexpressing eight genes encoding ET response factors, but overexpressing these genes did not lead to any change of plant size, except for plants overexpressing *ETHYLENE RESPONSIVE ELEMENT BINDING FACTOR22* (*ERF22*), which showed an increased plant size at 21 DAS. *Expansin* (*EXP*) and *Xyloglucan endotransglucosylases/hydrolase* (*XTH*) are known to be involved in loosening cell wall architecture and cell enlargement [37]. Upregulation of *XTH* genes has been observed in heterotic hybrids and hybrid mimic lines [17,38]. In this study, overexpression of *β-expansin 1* (*EXPB1*) induced increased plant size at 14 DAS, while overexpression of *EXPB3* did not change the plant size. Overexpressing *XTH19* showed variation of plant size, and the average plant size was decreased. *XTH19* showed tissue-specific expression, specifically in roots [39]. Constitutive *XTH19* expression in vegetative tissues may be negative for vegetative development. A growth–defence tradeoffs model has been proposed for heterosis or hybrid necrosis phenomena [1,40,41]. Overexpressing *CARBON/NITROGEN INSENSITIVE 1* (*CNI1)* or *Plant defensin 1.2b* (*PDF1.2b*) led to increased plant size at 21 DAS. *CNI1* is important for the carbon/nitrogen response during the early post-germinative growth, and overexpression of *CNI1* causes less sensitivity to change in C/N conditions [42]. *CNI1* expression was induced by *Pseudomonas syringae* pv. *tomato* DC3000 (*Pst*. DC3000) infection, and overexpression of *CNI1* increased resistance to *Pst*. DC3000 [43]. In addition, overexpression of *CNI1* suppressed the senescence phenotype [44]. *PDF1.2b* encodes plant defensin and is involved in non-host pre-invasive defence response [45]. Most transcriptome analyses comparing heterotic inter- or intra-hybrids and their parents have shown the downregulation of defence-responsive genes in F_1_ plants [14,17,33,46,47]. However, upregulation of some defence-response genes occurred in non-additively expressed genes, suggesting that upregulation of these genes might have a positive effect on plant growth. Overexpression of some genes, which were upregulated in big-sized F_1_ plants, showed an increased plant size at 14 or 21 DAS and a large variation of plant size within the lines. However, increased plant size was not observed at all time points. As plant size or heterosis is regulated by tissue- and stage-dependent transcriptional networks, a possible reason is that overexpression of a single gene is not enough to generate the phenotype, and overexpression of multiple genes may be required. Alternatively, changes in gene expression at particular time points may be important for increased plant size.

We found a variation in size in F_1_ plants of *A. thaliana* and identified genes that were differentially expressed between big- and medium-sized F_1_ plants. These differentially expressed genes tended to overlap with non-additively expressed genes in heterotic F_1_; however, increases and decreases in expression levels in big-sized plants/heterotic F_1_ did not always match. Non-additively expressed genes showed tissue and stage specificity [1]; e.g., upregulation of chloroplast-targeted genes was limited to a few days [14,18,19]. We suggest that changes in the expression of these genes, not the constitutively increased or decreased expression levels, may be important for increased plant size. Having variability in plant size in the F_1_ generation is not a suitable phenotype for F_1_ hybrid cultivars of crops, but this phenomenon may allow exploration of the factors necessary for maximizing the potential plant size or for stability of heterosis regardless of environmental effects.

## 4. Materials and Methods

### 4.1. Plant Materials and Growth Condition

F_1_ between C24 and Col accessions and its F_2_ population were used for analysis of plant size, microarray, and qPCR. Plants were grown in a controlled environment (22 °C) under fluorescent lights (150–180 μmol·m^−2^·s^−1^) and a 16-h/8-h (day/night) photoperiod. Plants were grown in plastic dishes containing Murashige and Skoog (MS) agar medium supplemented with 1.0% sucrose (pH 5.7) and were transferred to soil at 14 DAS.

We prepared 15 individual C24 (female) and Col (male) plants and crossed one combination each. We used the C24 line as female, and multiple flowers were used for making the F_1_ [14]. Using 13 of 15 F_1_ lines, we found a higher variation of rosette diameter at 14 DAS in C24xCol hybrids compared with parental lines (Figure S1). Furthermore, in this study, two F_1_ lines were selected for examination of variation of rosette diameter at 14 DAS, and one population was used for microarray analysis and the other population for qPCR. The other three F_1_ lines were used for examination of rosette diameter at 10 and 14 DAS and leaf area and cell size at 14 DAS.

F_2_ seeds were harvested from individual big- and medium-sized F_1_ plants.

### 4.2. Measuring Seed Size, Rosette Diameter, Leaf Area, and Cell Size

Dry mature seed was photographed under a stereoscopic microscope, and sizes were determined with Image-J software (http://rsb.info.nih.gov/ij/). Rosette diameter was measured for evaluation of plant size and equals the maximum diameter of the rosette as measured between the two largest leaves. Rosette diameter depends on leaf blade and petiole length. The first and second leaves at 14 DAS were fixed in a formalin/acetic acid/alcohol solution (ethanol/acetic acid/ formalin = 16:1:1). The leaf was photographed under a stereoscopic microscope, and sizes were determined with Image-J software. After examination of leaf area, they were cleared in a chloral hydrate/glycerol/water solution (chloral hydrate/H_2_O/glycerol = 8:2:1), and the samples were photographed under Nomarski optics. The palisade cell number per fixed unit area in the subepidermal layer of the centre of the leaf blade between the midvein and the leaf margin was counted. Three independent experiments were performed for examination of seed size, leaf area, and cell size. Statistical comparisons of seed size, leaf area, and cell size were performed using Student’s *t*-test (*p* < 0.05).

### 4.3. Expression Analysis

Total RNA was isolated from aerial tissues at 14 DAS in F_1_ or F_2_ plants using the SV Total RNA Isolation System (Promega). From 500 ng total RNA, first-strand cDNA was synthesized using random primers by SuperScript III Reverse Transcriptase (Invitrogen). Prior to qPCR, the specificity of the primer set for each gene was first tested by electrophoresis of PCR-amplified products using EmeraldAmp MAX PCR Master Mix (Takara bio) on 2.0% agarose gel in which single products were observed. Absence of genomic DNA contamination was confirmed by the PCR of no RT control. RT-PCR conditions were 95 °C for 3 min followed by 30 cycles of 95 °C for 30 s, 55 °C for 30 s, and 72 °C for 30 s. qPCR was performed using a Rotor-Gene 3000 Real-Time Cycler (Qiagen). The cDNA was amplified using Platinum Taq DNA polymerase (Invitrogen). PCR conditions were 95 °C for 2 min followed by 40 cycles of 95 °C for 30 s, 55 °C for 30 s, and 72 °C for 30 s. Expression levels of genes were calculated relative to *Isopentenyl pyrophosphate-dimethylallyl pyrophosphate isomerase 2* (*IPP2*) genes using the comparative quantification analysis method with Rotor-Gene 6 (Qiagen). Data presented are the average and standard error (s.e.) from two or three biological and three experimental replications. Primer sequences are shown in Table S8.

### 4.4. Microarray Analysis

Arabidopsis ATH1 Genome Array (Affymetrix) was used for transcriptome analysis. Total RNA (100 ng) from aerial tissues at 14 DAS from big- and medium-sized F_1_ plants was used for probe synthesis. Biotinylated cRNAs were synthesized using the IVT Labeling Kit (Affymetrix). Hybridization and scanning were performed according to the manufacturer’s instructions. Two independent biological replicates were performed. Data were analyzed following [14]. 

### 4.5. Gene Ontology Analysis

Analysis for enrichment of gene functional ontology terms was completed using the gene ontology (GO) tool agriGO [48]. The background reference for microarray analysis was the list of genes that displayed expression above-background in either the parental or F_1_ samples from each platform [14]. Statistical tests for enrichment of functional terms used the hypergeometric test and false discovery rate (FDR) correction for multiple testing to a level of 5% FDR.

### 4.6. Constructs and Plant Transformation

The complete coding sequence (CDS) were amplified by RT-PCR using gene-specific primers designed to add *Xba* I and *Sac* I or *Bam* HI and *Sac* I restriction sites to the 5′- and 3′-ends, and PCR products were cloned into pGEM T-easy vector (Promega). The DNA fragment was then inserted into *Xba* I and *Sac* I or *Bam* HI and *Sac* I restriction sites of the plant expression vector pBI121 under the control of CaMV35S promoter. The constructs were transformed into *Agrobacterium tumefaciens* strain EHA105, and transformation of Col accession was carried out by the floral dip procedure [49]. Positive transformants were selected in kanamycin (30 μg/mL) plates and confirmed by PCR. Primers used for constructing the vector are listed in Table S8. 

We selected T_2_ lines with a single transgene by segregation analysis using T_2_ plants with chi-square test. T_2_ plants were grown in plastic dishes containing MS agar medium supplemented with 1.0% sucrose (pH 5.7). At 14 DAS, they were transferred to soil and grown under long-day conditions (16 h light) at 22 °C. The presence or absence of transgene was examined by PCR using *neomycin phosphotransferase II* (*NPTII*) primer set (Table S8).

## Figures and Tables

**Table 1:** Number of genes overlapping with previous transcriptome data.

Transcriptome Data		B1&B2 > M1&M2 (361)		B1&B2 < M1&M2 (80)	
		Number	Percentage	Number	Percentage
C24/Col DEG	F_1_ > MPV (863)	102	28.3%	8	10.0%
	F_1_ < MPV (1,234)	53	14.7%	25	31.3%
C24/L*er* DEG	F_1_ > MPV (669)	114	31.6%	10	12.5%
	F_1_ < MPV (1,050)	47	13.0%	18	22.5%
Col/L*er* DEG	F_1_ > MPV (464)	21	5.8%	7	8.8%
	F_1_ < MPV (907)	63	17.5%	11	13.8%
C24xCol vs. *ddm1*C24x*ddm1*Col	WT > *ddm1* (73)	3	0.8%	0	0.0%
	WT < *ddm1* (1,128)	25	6.9%	0	0.0%
ColxC24 vs. *ddm1*Colx*ddm1*C24	WT > *ddm1* (69)	0	0.0%	4	5.0%
	WT < *ddm1* (1,208)	36	10.0%	0	0.0%

**Table 2:** List of genes for producing overexpressed transgenic plants.

Number	Gene Model	Name	Description	14 DAS ^#^	21 DAS ^#^
#1	At1g05575		Unknown protein	1.10**	0.88**
#2	At1g19210	*ERF17*	Encodes a member of the DREB subfamily A-5 of ERF/AP2 transcription factor family	1.00	0.95
#3	At1g22810	*ERF19*	Encodes a member of the DREB subfamily A-5 of ERF/AP2 transcription factor family	1.07	1.06
#4	At1g33760	*ERF22*	Encodes a member of the DREB subfamily A-5 of ERF/AP2 transcription factor family	1.08	1.04 *
#5	At2g26020	*PDF1.2b*	Predicted to encode a PR (pathogenesis-related) protein	1.04	1.14 *
#6	At2g35290	*SAUR79*	SMALL AUXIN UPREGULATED RNA 79	1.19 **	0.88 **
#7	At2g44840	*ERF13*	ETHYLENE-RESPONSIVE ELEMENT BINDING FACTOR 13	0.95	0.93
#8	At4g17490	*ERF6*	ETHYLENE RESPONSIVE ELEMENT BINDING FACTOR 6	1.06	1.02
#9	At4g30290	*XTH19*	XYLOGLUCAN ENDOTRANSGLUCOSYLASE/HYDROLASE 19	0.92 **	0.65 **
#10	At4g38840	*SAUR14*	SMALL AUXIN UPREGULATED RNA 14	1.01	0.99
#11	At5g07100	*WRKY26*	Encode WRKY DNA-binding protein 26	0.99	0.90
#12	At5g09570		Cox-19-like CHCH family protein	1.02	1.08
#13	At5g27420	*CNI1*	CARBON/NITROGEN INSENSITIVE 1	0.95	1.06 **
#14	At5g42380	*CML37*	Calmodulin like 37	1.16 **	0.85 **
#15	At5g47230	*ERF5*	ETHYLENE RESPONSIVE ELEMENT BINDING FACTOR 5	1.03	1.05
#16	At5g51190	*ERF105*	Encodes a member of the ERF subfamily B-3 of ERF/AP2 transcription factor family	1.02	0.93
#17	At3g02040	*GDPD1*	GLYCEROPHOSPHODIESTER PHOSPHODIESTERASE 1	0.94	1.00
#18	At3g23250	*MYB15*	MYB DOMAIN PROTEIN 15	1.22 **	0.91
#19	At4g34410	*RRTF1/ERF109*	REDOX RESPONSIVE TRANSCRIPTION FACTOR 1	1.03	0.92
#20	At2g20750	*EXPB1*	EXPANSIN B1	1.24 **	1.06
#21	At4g28250	*EXPB3*	EXPANSIN B3	0.98	0.98

^#^, ratio of rosette diameter in transgenic plants compared with non-transgenic plants; *, *p* < 0.05; **, *p* < 0.01 (Student’s *t*-test).

## Supplementary Materials

The following are available online at https://www.mdpi.com/2223-7747/9/4/414/s1, Figure S1: Distribution of rosette diameter at 14 DAS in parental lines and their reciprocal hybrids. Figure S2: Expression pattern of genes upregulated in big-sized F_1_ plants compared with medium-sized F_1_ plants and downregulated in *ddm1* F_1_ compared with wild-type F_1_. Figure S3: Plant phenotypes of transgenic plant lines of #9 and #19 at 21 DAS. Figure S4: Confirmation of transgene expression levels by RT-PCR. Table S1: Differentially expressed genes between big- and medium-sized F_1_ plants. Table S2: Genes showing differential expression between big- and medium-sized F_1_ plants and upregulation in C24/Col and C24/L*er* hybrids. Table S3: Validation of microarray expression data by quantitative RT-PCR. Table S4: Comparison of gene expression levels between big- and medium-sized F_2_ plants. Table S5: GO function term overrepresented in genes showing B1&B2 > M1&M2 expression. Table S6: GO function term overrepresented in genes showing B1&B2 < M1&M2 expression. Table S7: GO function term overrepresented in genes showing B1&B2 > M1&M2 expression and upregulation in both C24/Col and C24/L*er* hybrids. Table S8: Sequences of primers used in this study.
