# Supplementary material for: In Arabidopsis thaliana Heterosis Level Varies among Individuals in an F1 Hybrid Population
Source: Plants (Basel). 2020 Mar 27;9(4):414. doi: 10.3390/plants9040414 (PMC7238264; doi:10.3390/plants9040414)
Supplement: Supplementary file 1 [file plants-09-00414-s001.zip › Supplementary file/Supplementary figures.pptx]

## Slide 1
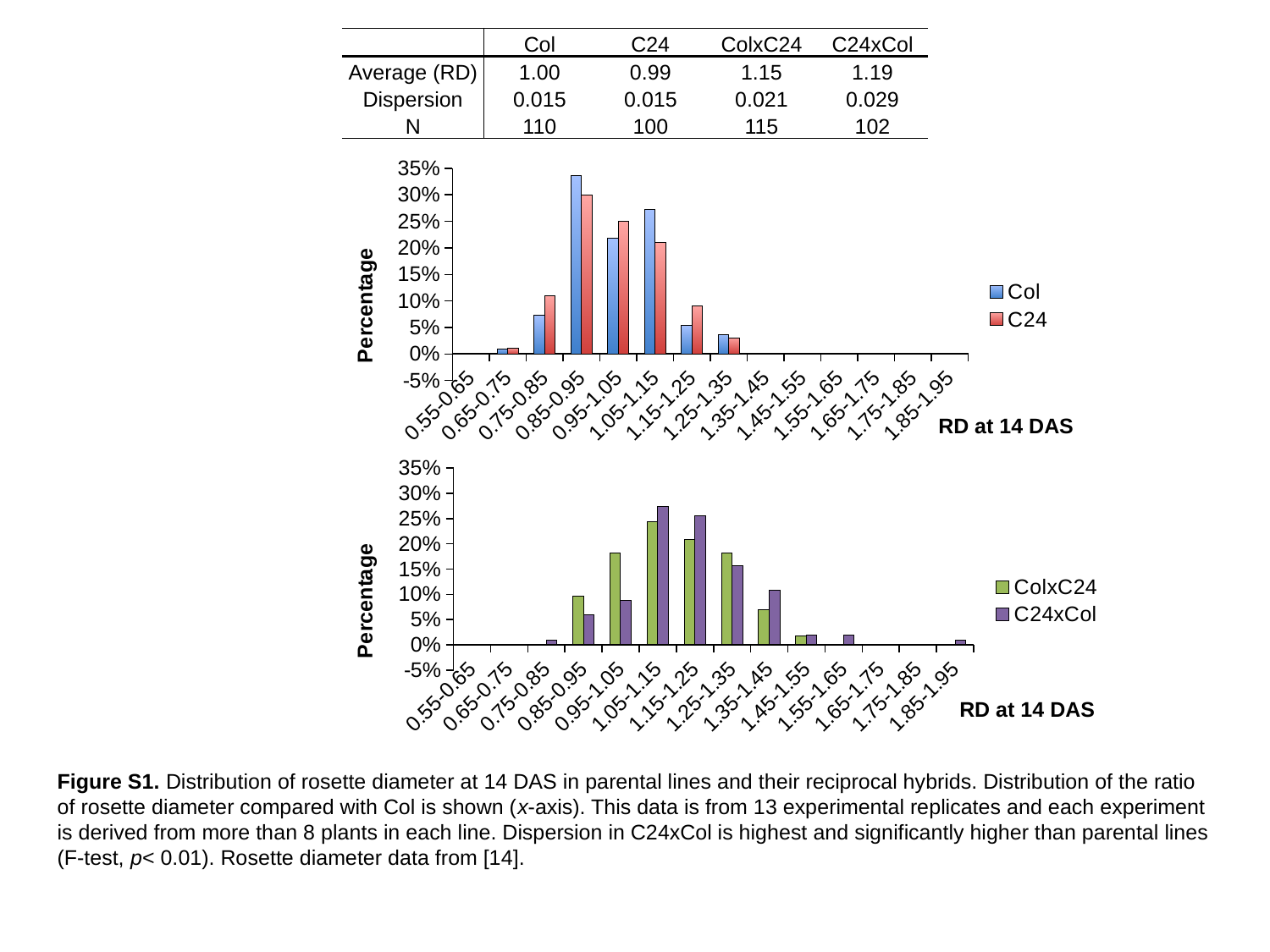

| | Col | C24 | ColxC24 | C24xCol |
| --- | --- | --- | --- | --- |
| Average (RD) | 1.00 | 0.99 | 1.15 | 1.19 |
| Dispersion | 0.015 | 0.015 | 0.021 | 0.029 |
| N | 110 | 100 | 115 | 102 |
### Chart
| Category | Col | C24 |
|---|---|---|
| 0.55-0.65 | 0.0 | 0.0 |
| 0.65-0.75 | 0.00909090909090909 | 0.01 |
| 0.75-0.85 | 0.0727272727272727 | 0.11 |
| 0.85-0.95 | 0.336363636363636 | 0.3 |
| 0.95-1.05 | 0.218181818181818 | 0.25 |
| 1.05-1.15 | 0.272727272727273 | 0.21 |
| 1.15-1.25 | 0.0545454545454545 | 0.09 |
| 1.25-1.35 | 0.0363636363636364 | 0.03 |
| 1.35-1.45 | 0.0 | 0.0 |
| 1.45-1.55 | 0.0 | 0.0 |
| 1.55-1.65 | 0.0 | 0.0 |
| 1.65-1.75 | 0.0 | 0.0 |
| 1.75-1.85 | 0.0 | 0.0 |
| 1.85-1.95 | 0.0 | 0.0 |RD at 14 DAS
### Chart
| Category | ColxC24 | C24xCol |
|---|---|---|
| 0.55-0.65 | 0.0 | 0.0 |
| 0.65-0.75 | 0.0 | 0.0 |
| 0.75-0.85 | 0.0 | 0.00980392156862745 |
| 0.85-0.95 | 0.0956521739130435 | 0.0588235294117647 |
| 0.95-1.05 | 0.182608695652174 | 0.088235294117647 |
| 1.05-1.15 | 0.243478260869565 | 0.274509803921569 |
| 1.15-1.25 | 0.208695652173913 | 0.254901960784314 |
| 1.25-1.35 | 0.182608695652174 | 0.156862745098039 |
| 1.35-1.45 | 0.0695652173913043 | 0.107843137254902 |
| 1.45-1.55 | 0.0173913043478261 | 0.0196078431372549 |
| 1.55-1.65 | 0.0 | 0.0196078431372549 |
| 1.65-1.75 | 0.0 | 0.0 |
| 1.75-1.85 | 0.0 | 0.0 |
| 1.85-1.95 | 0.0 | 0.00980392156862745 |RD at 14 DAS
Figure S1. Distribution of rosette diameter at 14 DAS in parental lines and their reciprocal hybrids. Distribution of the ratio of rosette diameter compared with Col is shown (x-axis). This data is from 13 experimental replicates and each experiment is derived from more than 8 plants in each line. Dispersion in C24xCol is highest and significantly higher than parental lines (F-test, p< 0.01). Rosette diameter data from [14].

## Slide 2
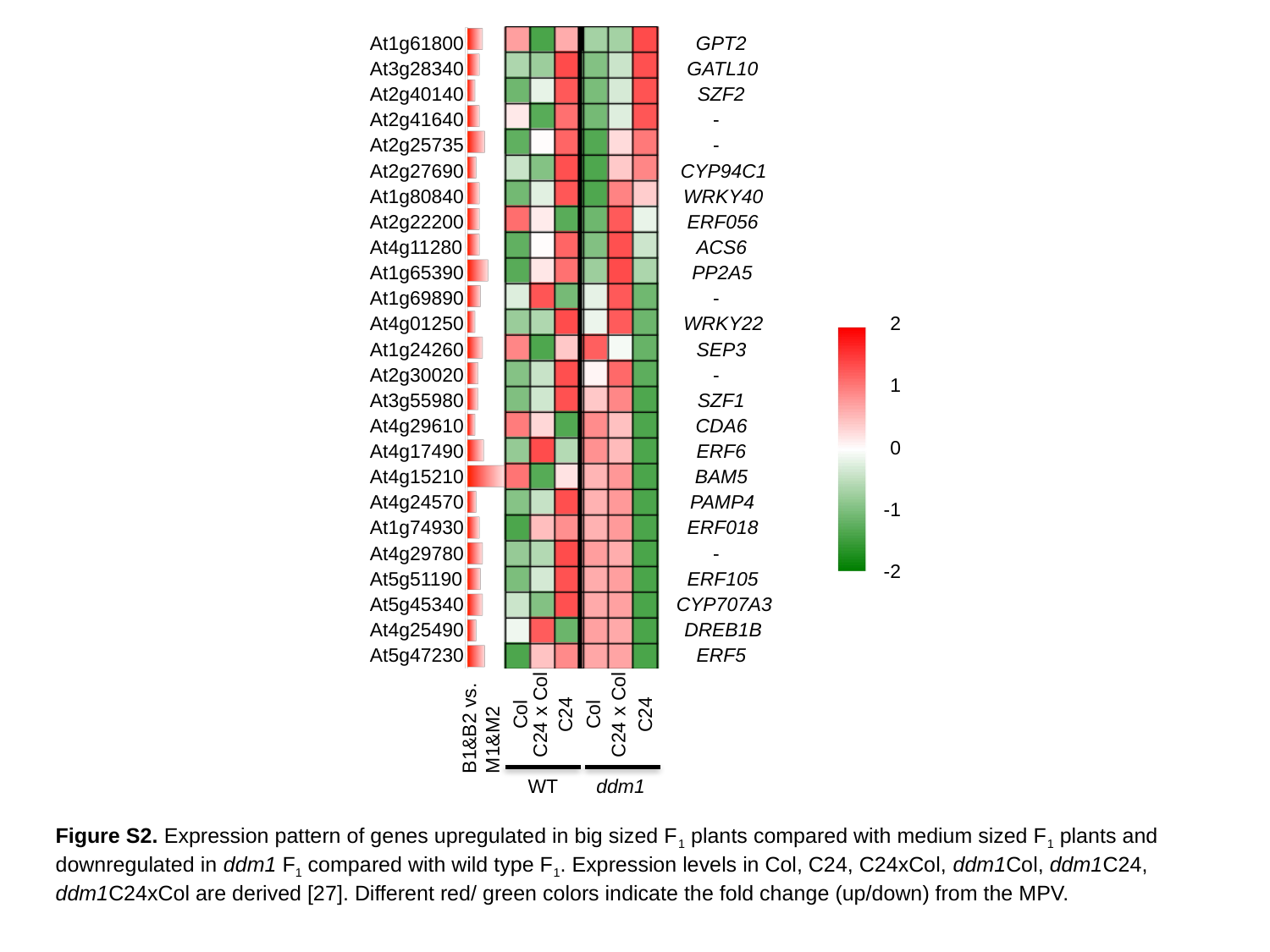

At1g61800
At3g28340
At2g40140
At2g41640
At2g25735
At2g27690
At1g80840
At2g22200
At4g11280
At1g65390
At1g69890
At4g01250
At1g24260
At2g30020
At3g55980
At4g29610
At4g17490
At4g15210
At4g24570
At1g74930
At4g29780
At5g51190
At5g45340
At4g25490
At5g47230
GPT2
GATL10
SZF2
-
-
CYP94C1
WRKY40
ERF056
ACS6
PP2A5
-
WRKY22
SEP3
-
SZF1
CDA6
ERF6
BAM5
PAMP4
ERF018
-
ERF105
CYP707A3
DREB1B
ERF5
2
1
0
-1
-2
B1&B2 vs.
M1&M2
C24 x Col
Col
C24
C24 x Col
Col
C24
WT
ddm1
Figure S2. Expression pattern of genes upregulated in big sized F1 plants compared with medium sized F1 plants and downregulated in ddm1 F1 compared with wild type F1. Expression levels in Col, C24, C24xCol, ddm1Col, ddm1C24, ddm1C24xCol are derived [27]. Different red/ green colors indicate the fold change (up/down) from the MPV.

## Slide 3
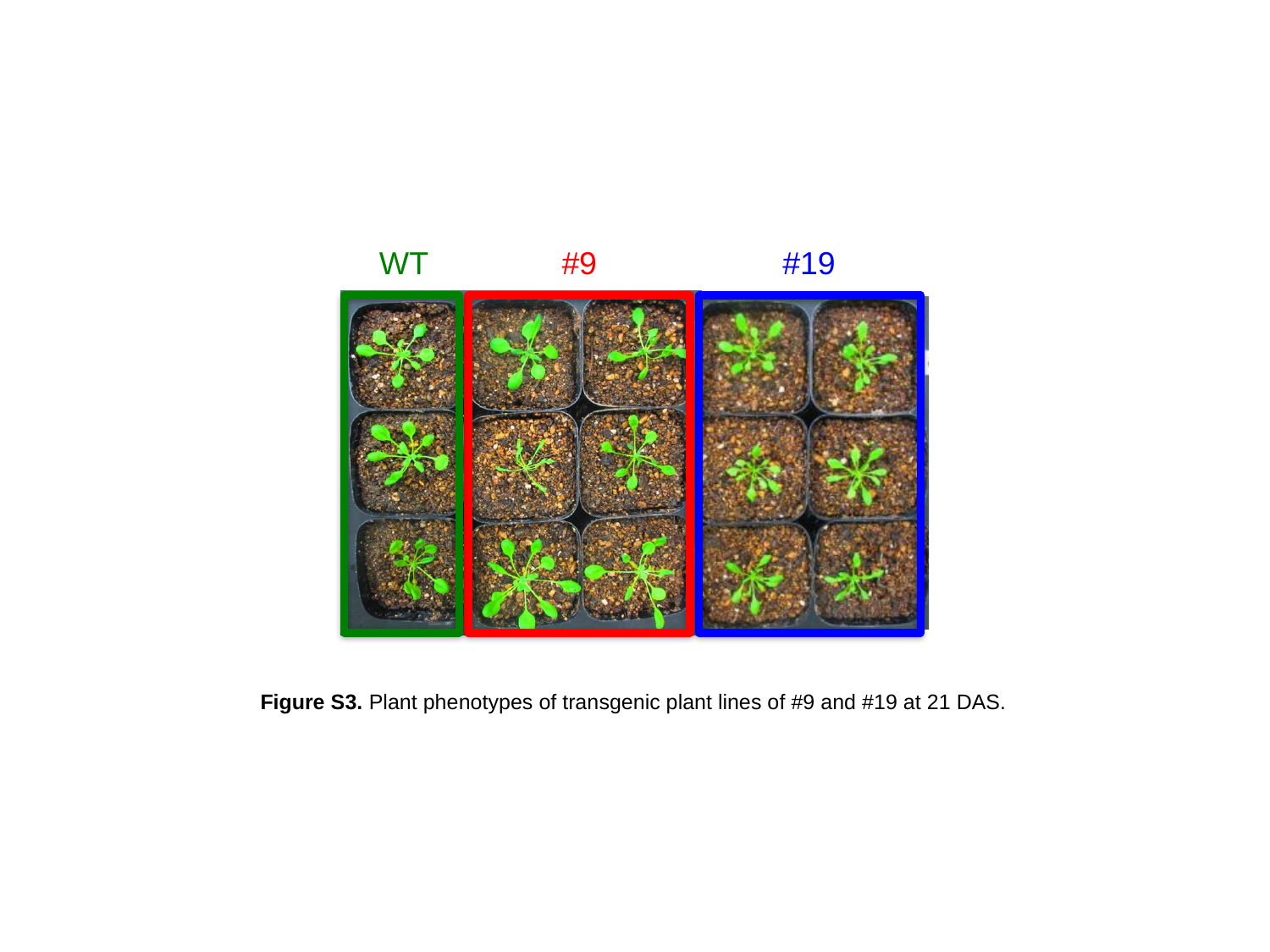

WT
#9
#19
Figure S3. Plant phenotypes of transgenic plant lines of #9 and #19 at 21 DAS.

## Slide 4
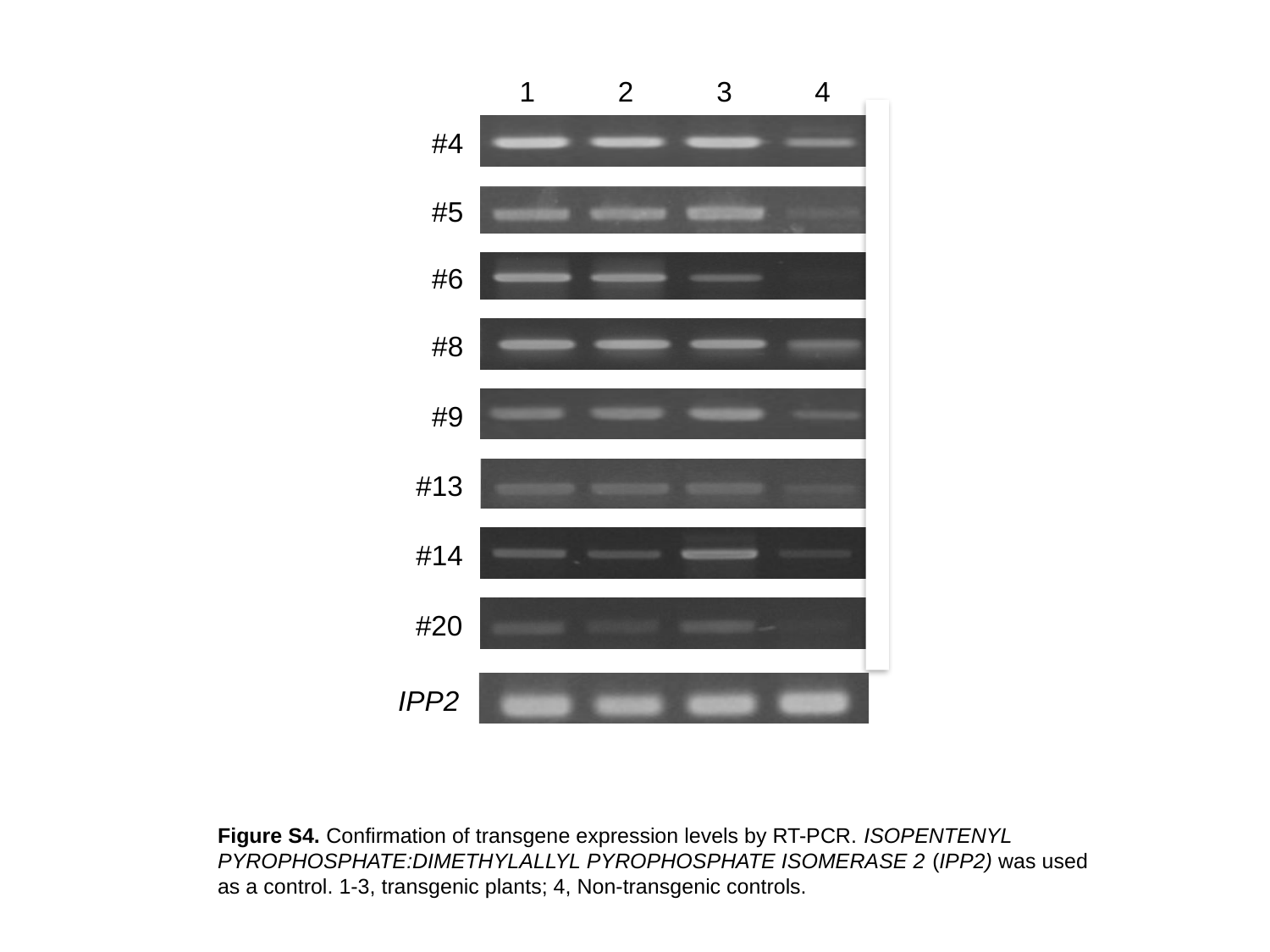

1
2
3
4
#4
#5
#6
#8
#9
#13
#14
 #20
IPP2
Figure S4. Confirmation of transgene expression levels by RT-PCR. ISOPENTENYL PYROPHOSPHATE:DIMETHYLALLYL PYROPHOSPHATE ISOMERASE 2 (IPP2) was used as a control. 1-3, transgenic plants; 4, Non-transgenic controls.
